# Unplanned medication discontinuation as a potential pharmacovigilance signal: a nested young person cohort study

**DOI:** 10.1186/2050-6511-15-11

**Published:** 2014-03-04

**Authors:** Angela Peichen Sun, Bradley Kirby, Corri Black, Peter John Helms, Marion Bennie, James Stuart McLay

**Affiliations:** 1 Division of Applied Health Sciences, University of Aberdeen, King's College, Aberdeen AB24 3FX, UK; 2 National Medicines Utilisation Unit, Information Services Division, NHS National Services Scotland, Edinburgh, Scotland; 3 Department of Child Health, Royal Aberdeen Children’s Hospital, Westburn Road, Aberdeen, Scotland AB25 2ZG, UK

**Keywords:** Pharmacovigilance, Pharmacoepidemiology, Obesity, Child, Orlistat, Adverse drug reaction, Medical records systems, Computerized, Young people

## Abstract

**Background:**

Because of relatively small treatment numbers together with low adverse drug reaction (ADR) reporting rates the timely identification of ADRs affecting children and young people is problematic. The primary objective of this study was to assess the utility of unplanned medication discontinuation as a signal for possible ADRs in children and young people.

**Methods:**

Using orlistat as an exemplar, all orlistat prescriptions issued to patients up to 18 years of age together with patient characteristics, prescription duration, co-prescribed medicines and recorded clinical (Read) codes were identified from the Primary Care Informatics Unit database between 1st Jan 2006-30th Nov 2009. Binary logistic regression was used to assess association between characteristics and discontinuation.

**Results:**

During the study period, 79 patients were prescribed orlistat (81% female, median age 17 years). Unplanned medication discontinuation rates for orlistat were 52% and 77% at 1 and 3-months. Almost 20% of patients were co-prescribed an anti-depressant. One month unplanned medication discontinuation was significantly lower in the least deprived group (SIMD 1–2 compared to SIMD 9–10 OR 0.09 (95% CI0.01 – 0.83)) and those co-prescribed at least one other medication. At 3 months, discontinuation was higher in young people (≥17 yr versus, OR 3.07 (95% CI1.03 – 9.14)). Read codes were recorded for digestive, respiratory and urinary symptoms around the time of discontinuation for 24% of patients. Urinary retention was reported for 7.6% of patients.

**Conclusions:**

Identification of unplanned medication discontinuation using large primary care datasets may be a useful tool for pharmacovigilance signal generation and detection of potential ADRs in children and young people.

## Background

Although adverse drug reactions (ADR) represent a major source of morbidity and mortality in adults, the nature and frequency of ADRs affecting children and young people remains poorly defined. Published data suggest that ADRs account for 1.5-2.1% of paediatric hospital admissions and affect 2.6-9.3% of paediatric inpatients and 1.5-11.1% of paediatric outpatients [1-10].

In the past children and young people have frequently been excluded from clinical trials and where trials are undertaken, they are often insufficiently powered to detect even relatively common ADRs. This means that children and young people are often prescribed medication with limited information about a treatment’s ADR profile, at doses which are not evidence based, and “off–label”; a practice which itself is associated with increased risk of ADRs [2].

Post marketing surveillance is an essential tool in enabling the detection of potentially serious ADRs; however, to detect an ADR with a frequency of 1:10,000, at least 30,000 individuals need to be treated with the drug for at least 1 case to be identified. Therefore, post-marketing surveillance requires the monitoring of large patient cohorts exposed to a medication to detect ADRs. Currently, pharmacovigilance systems rely primarily on spontaneous reporting of ADRs. However under-reporting of ADRs is a significant issue affecting all European ADR monitoring systems. It is widely accepted that only 10% of UK and European healthcare professionals take part in the process of ADR reporting and that only 6-9% of ADRs affecting adults, which should be reported, are actually reported to the regulatory authorities [11-15]. Despite extending spontaneous ADR reporting in the UK to the public, and a wider group of healthcare professionals, public reporting rates still remain low, and significantly lower for children and young people than adults [16].

A growing area of medical concern and drug treatment is that of obesity [17,18]. Obese adolescents tend to be become obese adults, and it is well recognised that obesity in adults is associated with increased morbidity and mortality [19,20].

In the UK, orlistat, is currently the only anti-obesity drug licensed for use in severely obese (Body Mass Index, BMI ≥30 kg/m^2^) adult patients or obese patients with comorbidities. Use in adults is associated with a 30% discontinuation within 3 months due to intolerable ADRs [21-24]. In pediatric/adolescent clinical trials, orlistat use has also been associated with a high level of ADRs (97-100%) and medication discontinuation (3.4-30%). However the majority of these orlistat studies have included less than 30 subjects so the ADR profile in children and young people is poorly understood [25-27].

In the UK, the majority of children and young people who require medical intervention for obesity, even when assessed in secondary care, are routinely reviewed and prescribed their medication in primary care. Primary Care electronic administrative data provide a record of relevant clinical information, service contacts and prescribing information and can, therefore, be used to assess the effectiveness of medications and potentially to generate pharmacovigilance signals. Such datasets which have been previously used for pharmacoepidemiology t have not been widely used for pharmacovigilance and have the potential to offer a complementary approach to routine pharmacovigilance methodologies.

Unplanned medication discontinuation (UMD), as a signal for the identification of possible ADRs, has been previously assessed in the adult population using Primary Care electronic administrative data. This approach is based on the recognition of an early, unplanned discontinuation of the prescription for a medication, possibly accompanied by switching to alternative medication. A close agreement has been previously reported between the UMD rate and ADR rates reported in the literature for a variety of adult medications [28].

The primary objective of this study was to assess the utility of UMD as a signal for possible ADRs in children and young people. Using a large Primary Care electronic administrative dataset and orlistat as an exemplar, we assessed prescribing pattern, medicine use and the potential to generate pharmacovigilance signals for medicines prescribed with low frequency to special populations such as children and young people.

## Methods

### Data source

The Primary Care Clinical Informatics Unit (PCCIU) research database is a large administrative dataset covering, in 2006, 179 Primary Care practices across Scotland [29]; representing approximately 20% of the Scottish population (1 million patients).

The PCCIU database, contains demographic data including gender, date of birth and socioeconomic status based on the practice post code (the Scottish Index of Multiple Deprivation (SIMD) 2006 quintiles) [30]: clinical information including diagnoses, procedures and practice encounter data, all recorded as Read codes, and prescribing data. Read codes are a national coding system used in primary care to code and record relevant information arising from a patient encounter in a standardised format. Read codes permit codification of a patient’s history, symptoms, examination findings, physical signs, diagnostic procedures, therapeutic and administrative procedures, drugs, appliances, occupation and social information [31]. PCCIU contains validated clinical data on a large representative proportion of the Scottish population [30,32,33].

### Study population

The study population included a closed cohort, all children and young people aged 0–16 years of age registered with a General Practice on the 1st January 2006. This cohort was then followed until they reached their 19th birthday or 30th November 2009.

### Exposure

All children and young people newly prescribed orlistat (British National Formulary code 4.5) were identified and tracked through the study period.

For this study, a “new prescription” was defined as the first prescription for orlistat during the study period with no previous prescription in the preceding 3 months.

### Primary outcome

The primary outcome of interest was identification of a potential pharmacovigilance signal, defined as the discontinuation of orlistat for at least 3 months without a subsequent prescription. Unplanned medication discontinuations were categorised based on duration of treatment as: <1 month, 1–3 months, 4–6 months or >6 months. Duration was calculated from the date of the first prescription to the date of the last prescription. If prescriptions were ongoing at the end of the study, the child was categorised as continuing treatment.

Because the majority of patient records identified in the PCCIU dataset did not contain a specific reason for drug discontinuation, clinical symptoms, diagnoses, investigations and procedures recorded as Read codes around the time of medication discontinuation were examined. All Read codes occurring after starting orlistat were identified. Potential signal associations were limited to codes recorded during the 3 months before and 3 months after the last prescription. Read codes were reported and summarised according to the International Classification of Diseases (ICD)-10 top level categories.

### Other covariates

All co-prescriptions were extracted from the date of the first orlistat prescription up to 3 months after the last orlistat prescription date and classified into their respective British National Formulary (BNF) therapeutic drug classes [33]. The number of therapeutic drug classes prescribed for each patient was then determined and classified into 3 groups: no co-prescriptions issued, 1–4 therapeutic drug classes prescribed and >4 drug classes prescribed.

As national guidelines recommend that the BMI be calculated for the purposes of diagnosis, monitoring and initiation of pharmacological treatment, the date of the last recorded weight and height was also noted, and if assessed more than 1 year before start of orlistat was treated as missing.

To allow comparison between gender and developmental stages, the BMI standard deviation score (BMI SDS) was calculated based on Cole’s LMS method [34].

Individuals with a BMI SDS between 2.5 – 3.49 were classified as overweight to obese, those with a BMI SDS over 3.5 classified as severely obese and those over 4 as very severely obese.

### Statistical analysis

Descriptive statistics (percentages, mean (SD), median (IQR)) were used to report the characteristics of the cohort. The incidence rate for orlistat prescription was calculated for 2006.

Discontinuations were summarised as percentages and reported graphically.

To explore factors that might influence the discontinuation of orlistat treatment, we compared those who discontinued orlistat treatment at one and three months with those continuing treatment. We used binary logistic regression to calculate unadjusted odds ratios (OR) and 95% confidence intervals (CI). The factors considered included: gender, age, socioeconomic status, co-prescribing of antidepressants, and number of co-prescriptions. Analysis was performed using SPSS for windows 19.0 (SPSS Inc, Chicago, Illinois, USA).

### Ethics and approvals

Data were anonymised and no personally identifiable information was sought. Approval for the study was granted by the Primary Care Clinical Informatics Unit research steering committee in accordance with their research governance process.

## Results

From the study population of 166,726 children and young people (49% female) aged 0–16 years and registered with a participating primary care practice in Jan 2006, a cohort of 79 young people newly prescribed orlistat during the study period were identified. In 2006, at cohort inception, the incidence of orlistat was 0.06/1000/year for children aged 0–16 years.

### Characteristics of the individuals prescribed orlisat

The median age at first orlistat prescription was 17 years (IQR 16 – 18), 81% were female and 55% were from General Practices situated in the most deprived socioeconomic areas (SIMD deciles 7–10) (Table 1). Of the 79 children and young people prescribed orlistat, only 54 (68.3%) had a BMI recorded, of which 42 (53.2%) had a BMI measured within 1 year, and 28 (35.4%) within one month of orlistat initiation. For the 42 individuals in whom BMI was measured within a year of orlistat initiation, the median time between BMI measurement and the index orlistat prescription was 0.3 months (IQR 0.0 – 2.2 months). The mean BMI SDS was 3.31 (SD0.9) with only 16 (20.3%) of all patients prescribed orlistat having a recorded BMI SDS greater than 3.5. The demographic characteristics of individuals prescribed orlistat is described in Table 1.

**Table 1:** Patient characteristics at time of first orlistat prescription during the follow up period (1st Jan 2006 -30th Nov 2009)

	**Orlistat population**	**Male**	**Female**
	**(N = 79)**	**(N = 15)**	**(N = 64)**
		**(19% of total)**	**(81% of total)**
**Age** n (%)			
Children (under 17 years)	24 (30.5%)	4 (26.7%)	20 (31.3%)
Young people (17 – 18 years)	55 (69.6%)	11 (73.3%)	44 (68.7%)
*Median (IQR)*	*17 (16–18)*		
**BMI SDS** n (%)			
<2.5	12 (15.2%)	0	12 (18.8%)
2.5 – 3.49	24 (30.4%)	6 (40%)	18 (28.1%)
>3.5	18 (22.8%)	6 (40%)	12 (18.8%)
Missing	25 (31.6%)	3 (20%)	22 (34.4%)
*Mean (SD)*	*3.13 (1.0)*		
**Deprivation SIMD (2006)** n (%)			
1-2 (least deprived)	8 (10.1%)	1 (6.7%)	7 (10.9%)
3-4	7 (8.9%)	2 (13.3%)	5 (7.8%)
5-6	20 (25.3%)	3 (20.0%)	17 (26.6%)
7-8	18 (22.8%)	7 (46.7%)	11 (17.2%)
9-10 (most deprived)	26 (32.9%)*	2 (13.3%)	24 (37.5%)
**Co-prescribed drug classes** n (%)			
0	24 (30.4%)	7 (46.7%)	17 (26.6%)
1-4	52 (65.8%)	8 (53.3%)	44 (68.8%)
>4	3 (3.8%)	0 (0.0%)	3 (4.7%)
*Median (IQR)*	*1 (0–2)*		

All percentage calculated from column total. *Chi squared test for trend p = 0.03.

SIMD: Scottish Index of Multiple Deprivation, IQR: Inter-quartile range, BMI SDS: Body mass index standard deviation score.

Two thirds (65.8%) of patients were co-prescribed between 1–4 drug classes/items. The medicines most commonly co-prescribed were antibiotics (17.4% of all prescriptions), topical skin treatments (12.4%), analgesics (10.7%), oral contraceptives (9.9%), asthma medications (7.4%) and antidepressants (4.1%). Four (5%) patients prescribed orlistat were also prescribed metformin. For three of these, metformin was initiated immediately following cessation of the orlistat prescription.

### Orlistat discontinuation

Fifty-two percent of patients (41) discontinued orlistat medication within 1 month of the index prescription and 77% (61) within 3 months.

Discontinuation within 1 month was not affected by age or gender but was significantly less likely if the patient had received any co-prescription (OR 0.12 (95% CI 0.03 – 0.41) or was in the least deprived group (OR 0.09 (95% CI 0.01 – 0.84) (SIMD 1–2) though there was no obvious trend by deprivation category.

Gender, co-prescription and socio-economic status, were not significantly related to 3-month discontinuation, however young people over 17 years of age were three times more likely to discontinue their prescription at 3-months when compared with those under 17 years of age (OR 3.07; 95%Cl 1.03 – 9.14) (Table 2).

**Table 2:** Crude odds ratios for 1 month and 3 month discontinuation for factors such as: gender, age, deprivation score, prescription of anti-depressants and co-prescription of other therapeutic drug classes

	**Duration of treatment**				**Duration of treatment**			
	**<1 month**	**≥1 month**	**OR**	**95% CI**	**<3 month**	**≥3 month**	**OR**	**95% CI**
	**N = 41**	**N = 38**			**N = 61**	**N = 18**		
**Gender***n (%)*								
Female	33 (80.5%)	31 (81.6%)	Ref		48 (78.7%)	16 (88.9%)	Ref	
Male	8 (19.5%)	7 (18.4%)	1.07	0.35–3.31	13 (21.3%)	2 (11.1%)	2.17	0.44– 10.65
**Age bands***n (%)*								
Children (under 17 years)	9 (22.0%)	15 (39.5%)	Ref		15 (24.6%)	9 (50.0%)	Ref	
Young people (17-18 years)	32 (78.0%)	23 (60.5%)	2.32	0.87–6.21	46 (75.4%)	9 (50.0%)	3.07	1.03– 9.14
**Deprivation SIMD 2006***n (%)*								
1-2	1 (2.4%)	7 (18.4%)	0.09	0.01–0.84	5 (8.2%)	3 (16.7%)	0.23	0.03-1.41
3-4	3 (7.3%)	4 (10.5%)	0.47	0.09–2.55	6 (9.8%)	1 (0.6%)	0.78	0.07– 8.93
5-6	12 (29.3%)	8 (21.1%)	0.94	0.28–3.09	15 (24.6%)	5 (27.8%)	0.39	0.08– 1.89
7-8	9 (22.0%)	9 (23.7%)	0.63	0.19–2.11	12 (19.7%)	6 (33.3%)	0.26	0.06– 1.23
9-10	16 (39.0%)	10 (26.3%)	Ref		23 (37.7%)	3 (16.7%)	Ref	
**Antidepressants***n (%)*								
Yes	37 (90.2%)	28 (73.7%)	Ref		51 (83.6%)	14 (77.8%)	Ref	
No	4 (9.8%)	10 (26.3%)	0.30	0.09–1.07	10 (16.4%)	4 (22.2%)	0.69	0.19– 2.52
**Co-prescriptions issued***n (%)*								
Yes	20 (48.8%)	4 (10.5%)	Ref		22 (36.1%)	2 (11.1%)	Ref	
No	21 (51.2%)	34 (89.5%)	0.12	0.03–0.41	39 (63.9%)	16 (88.9%)	0.22	0.05– 1.06

*CI; Confidence Interval. OR; Odds Ratio*.

All percentage calculated from column total. SIMD: Scottish Index of Multiple Deprivation.

### Read codes and possible adverse events

In total, 404 Read codes were recorded from the start of orlistat treatment to 3 months after the last orlistat prescription for 77.2% (61) of the study population. The median number of Read codes per individual was 5; IQR 1–6 (range 0–51), and 20.3% (82) of these Read codes related specifically to symptoms or treatments. Eight (10.1%) patients had a Read Code recorded for: the respiratory, seven (8.9%) for the genitourinary, six (7.6%) for skin, six (7.6%) for endocrine, four (5.1%) for obstetric and gynaecological, four (5.1%) for gastrointestinal, four (5.1%) for musculoskeletal, 3 (3.8%) for mental health and behavioural and 3 (3.8%) for the CNS systems. (Symptoms and diagnoses are listed in Table 3).

**Table 3:** Actual Symptoms and diagnoses recorded as Read codes by the treating primary care physician within 3 months of orlistat discontinuation (n = 79)

**System**	**Number of patients (%)**	**Symptoms**	**Frequency**
Respiratory	8 (10.1%)	Influenza	3
		Acute tonsillitis	3
		Acute bronchitis and bronchiolitis	2
		Asthma	1
		Chest infection	1
		Acute laryngitis and tracheitis	1
		Upper respiratory infection	1
		Cough	1
		Respiratory symptom	1
Genitourinary	7 (8.9%)	Treatment via bladder catheter	5
		Retention of urine	1
		Urinary tract infection (UTI)	1
		Suspected UTI	2
		Cystitis	1
Dermatology	6 (7.6%)	Rash and other non-specific skin eruption	1
		Dermatitis	1
		Skin cyst	1
		Chalazion (meibomian cyst)	1
		Acne vulgaris	1
		Unilateral mastalgia	1
		Lump in breast	1
		Psoriasis and similar disorders	1
Endocrine	6 (7.6%)	Obesity	4
		Acquired hypothyroidism	2
		Gynaecomastia	3
Obstetrics & Gynaecology	4 (5.1%)	Menorrhagia	1
		Dysmenorrhoea	1
		Spontaneous abortion	1
		Codes related to spontaneous abortion	6
		Polycystic ovarian syndrome	1
Gastrointestinal	4 (5.1%)	Non-infective gastritis	1
		Gastritis	1
		Abdominal pain	3
		Abdominal discomfort	1
		Irritable bowel syndrome	1
Musculoskeletal	4 (5.1%)	Backache	1
		Pain in lumbar spine	1
		Closed fracture of radius	1
		Temporomandibular joint disorder	1
		Arthralgia of knee	1
Mental health and behavioural	3 (3.8%)	Social phobias	4
		Loss of confidence	1
		Anxiety state	1
		Depressed mood	4
		At risk of deliberate self harm	1
Central nervous system	3 (3.8%)	In-coordination	1
		Tiredness	1
		Migraine	1
		Headache	1
		Infantile autism	1
Infection	3 (3.8%)	Viral infection	3

Of note 8.9% (7, all female) of all patients prescribed orlistat had a Read code recorded within three months of orlistat discontinuation for the genitourinary system, of whom five were recorded as having urinary catheterisation and one urinary retention. None of these individuals were prescribed any medication or items during the three year study period to suggest neurological, bowel, bladder or malignant disease, which might explain the need for urinary catheterisation.

## Discussion

We demonstrate that using UMD as a surrogate for possible adverse events, routinely collected primary care datasets may be used for pharmacovigilance signal generation, although validation of generated signals and confirmation of potential ADRs will require appropriately designed prospective studies. We believe that this approach will be complementary to the spontaneous reporting systems currently in use and enhance the capability of these systems to widen the breadth of ADRs detected while reducing cost and time to ADR detection. Furthermore such an approach permits assessment of prescribing appropriateness together with real life prescribing patterns. In this study, one in every two patients prescribed orlistat discontinued within a month, and more than three quarters discontinued within three months, of starting treatment.

For medicines such as orlistat, continued use is likely to reflect patient acceptability and perceived effectiveness. In this context it is likely that early discontinuation reflects the appearance of unwanted poorly tolerated ADRs, while later discontinuation represents a perceived lack of response [35]. The levels of UMD in this study are similar to those reported by Viner at al, who noted orlistat discontinuations of 45% at one month, 75% at 3 months and 90% at 6 months [36].

Discontinuation at three months was significantly more frequent in the young people aged 17 years and over, possibly reflecting the effects of emerging autonomy and loss of parental influence [37-39]. One month discontinuation was significantly associated with social class and the presence of other co-prescribed medication, with patients from the most affluent social groups and those co-prescribed other medications less likely to discontinue orlistat therapy. While the association between social deprivation and poor adherence is well recognised the observation that medication discontinuation was less likely in those co-prescribed other medications runs counter to the perceived wisdom that higher levels of non-adherence are observed among adolescents and young adults prescribed multiple medicines [40,41].

In this study orlistat was prescribed predominantly to adolescents females, and those from the most socially deprived areas. Although the relationship between obesity and social deprivation is well recognised, this is the first study to report a link between social deprivation and orlistat prescribing [42-46].

Seventy percent of the cohort prescribed orlistat was co-prescribed medications including antibiotics, topical treatments for skin conditions and analgesia. Five percent of patients were also prescribed metformin, licensed for use in individuals aged 10 years and older for type II diabetes mellitus, but which is being increasingly used for the treatment of obesity and insulin resistance [46-51].

In the present study, one fifth of all patients (predominantly female) prescribed orlistat were also prescribed antidepressants at some point during the study period. In the general adolescent population antidepressants are reportedly prescribed to 1-5/1000 individuals per year [52-54], therefore the high level of use observed in our study suggests a significantly greater prevalence of depression in the obese t population a finding consistent with studies in both young people and adults [54-56].

Read code, describing clinical symptoms and treatments, were recorded by a primary care physician for approximately a fifth of patients around the time of orlistat discontinuation. Respiratory, genitourinary, endocrine, skin, gynaecological and gastrointestinal symptoms were reported to occur in 10%, 8.9%, 7.6%, 7.6%, 5.1% and 5.1% of patients respectively. Data from pediatric/young person orlistat trials suggest that adverse effects, primarily diarrhoea, affect 97-100% of individuals, leading to a discontinuation rate of 2% - 30% [26,27,57]. In this study a Read code for gastrointestinal symptoms (all of which were abdominal pain) was only recorded for 5.1% of patients. The reasons for this apparent difference between our data and that from clinical trials may be that patients were pre-warned about possible gastrointestinal symptoms and therefore did not report the ADR, or that gastrointestinal side effects were so common that primary care physicians did not record them as a reason for discontinuation.

An unexpected finding was the number of individuals for whom urological system Read codes, urinary catheterisation and urinary retention, were recorded. The reasons for this finding are not clear. While urinary retention is frequently associated with neurological disorders and or constipation, assessment of co-prescribed medicines and items for these individuals, identified only one subject prescribed laxatives and none prescribed medicines or items commonly used for the treatment of neurological, bowel, bladder, malignant or infective conditions during the study period. Although orlistat use has been associated with nephrolithiasis and acute renal injury [58,59], there are currently no data in the literature linking orlistat with urinary retention. However as of November 2013 (current reporting period), the Medicines and Healthcare products Regulatory Agency, the UK government medicine regulatory agency, had received two “Yellow Card” reports for urinary retention possibly associated with orlistat use [60], raising the possibility that the association between orlistat use and urological symptoms observed in this study may be real. Although the approach used in this study doesn’t allow us to attribute causality, it is reassuring to observe that the symptoms and diagnoses recorded by primary care physicians around the time of orlistat discontinuation, such as influenza infection, upper respiratory infection, headache and menstrual irregularities are in close agreement with those reported for adults in the real world [61].

Current clinical guidelines make it clear that prior to the initiation of orlistat therapy, height and weight should be recorded. In our study, 70% of the study cohort had this information recorded, and of these only a third had measurements recorded within a month of their initial prescription. The failure to undertake appropriate BMI assessment prior to orlistat initiation means that it is not possible to determine for the majority of patients whether orlistat was prescribed appropriately, according to the UK guidelines.

We have previously reported that electronic prescribing databases can be used to identify UMD in adult patients, and that such UMD rates can be used as a surrogate for ADR/AE rates [28,35]. However, the use of UMD does not identify the reasons for discontinuation or the nature of possible ADRs/AEs. The UMD and ADR results we obtained for orlistat are in close agreement with previously published UMD and ADR rates obtained using more traditional methods of ascertainment, indicating that UMD obtained from routinely collected data may represent a surrogate marker for ADR/ADE occurrence. Using this approach together with more traditional spontaneous reporting systems [62], may reveal potential pharmacovigilance signals at an earlier stage following introduction of a medicine and permit a more accurate assessment of ADR incidence than is currently possible.

The use of large primary care electronic datasets enables the linkage of whole population prescription information with recorded clinical symptoms, diagnoses and encounters. As we have demonstrated, this approach permits the assessment of prescribing trends and identification of individuals who discontinue their medication at an early stage before clinical effectiveness might be expected. Assessment of clinical symptoms and diagnosis recorded around the time of orlistat discontinuation identified unexpected findings which might indicate, although not prove, the occurrence of an ADR to the study medication.

There were several limitations to our study. It is possible that the small number of patients prescribed orlistat in this study may reduce the generalisability of the orlistat specific findings; however it does not alter the overall conclusion that the linkage of UMD with medical Read codes identifies possible pharmacovigilance signals of interest. Moreover, the PCCIU-R database, like most other prescribing/dispensing databases, only reflects primary care prescribing and does not provide information on secondary care prescribing or over the counter purchase of orlistat. It is therefore possible that patients for whom an initial prescription was issued in secondary care or who purchased their medication OTC may have been missed. However this is unlikely to be a major issue in the Western world, where primary care remains the major source of medication prescriptions. A further limitation is the lack of a specific recorded reason for orlistat discontinuation in the PCCIU-R database, and this is the reason why all Read codes at or about the time of discontinuation were identified and examined.

## Conclusions

Using orlistat as the exemplar, we have demonstrated that when unplanned medication discontinuation are linked to clinical codes for events occurring at the time of discontinuation, it is possible to generate both predictable and unpredicted pharmacovigilance signals. These findings support the use of this approach together with traditional pharmacovigilance methodologies to identify possible signals and plan more specific and focused pharmacovigilance studies with the aim of validating suspected ADRs.

### Patient consent

As all data analyzed was fully anonymised and non attributable the approval for the study was granted by the Primary Care Clinical Informatics Unit research steering committee in accordance with their research governance process and individual patient consent was not necessary.

## Abbreviations

ADR: Adverse drug reaction; BMI: Body mass index; BMISDS: Body mass index standard deviation score; BNF: British National Formulary; ICD: International Classification of Diseases; MHRA: Medicines and healthcare products regulatory agency; PCCIUR: Primary care clinical informatics unit research; NICE: National institute for health and clinical excellence; SIMD: Scottish index of multiple deprivation; SIGN: Scottish intercollegiate guidelines network.

## Competing interests

The authors declare that they have no competing interests.

## Authors’ contributions

AS: assisted in conceptualization, design and analysis of the study, drafted the initial manuscript, and approved the final manuscript as submitted. BK: assisted in conceptualization, design and analysis of the study and approved the final manuscript as submitted. PH: assisted in conceptualization, design and analysis of the study and approved the final manuscript as submitted. CB: assisted in conceptualization, design and analysis of the study and approved final manuscript as submitted. MB: assisted in conceptualization, design and analysis of the study and approved the final manuscript as submitted. JM: conceptualized and designed the study, assisted with analysis and interpretation of results, and approved the final manuscript as submitted. JM acts as study guarantor. All authors read and approved the final manuscript.

## Pre-publication history

The pre-publication history for this paper can be accessed here:

http://www.biomedcentral.com/2050-6511/15/11/prepub
